# What Kinds of Computations Can Young Children Perform Over Non-Symbolic Representations of Small Quantities?

**DOI:** 10.1162/opmi_a_00177

**Published:** 2025-01-04

**Authors:** Chen Cheng, Melissa M. Kibbe

**Affiliations:** Division of Social Science, The Hong Kong University of Science and Technology, Hong Kong, China; Department of Psychological and Brain Sciences, Boston University, Boston, MA, USA

**Keywords:** parallel individuation system, non-symbolic arithmetic, addition, addend unknown, preschool

## Abstract

Children can manipulate non-symbolic representations of both small quantities of objects (about four or fewer, represented by the parallel individuation system) and large quantities of objects (represented by the analog magnitude system, or AMS). Previous work has shown that children can perform a variety of non-symbolic operations over AMS representations (like summing and solving for an unknown addend), but are not able to perform further operations on the derived solutions of such non-symbolic operations. However, while the computational capacity of AMS has been studied extensively in early childhood, less is known about the computational capacity of the parallel individuation system. In two experiments, we examined children’s ability to perform two types of arithmetic-like operations over representations of small, exact quantities, and whether they could subsequently perform novel operations on derived quantity representations. Four-6-year-old US children (*n* = 99) solved two types of non-symbolic arithmetic-like problems with small quantities: summation and unknown addend problems. We then tested whether children could use the solutions to these problems as inputs to new operations. Results showed that children more readily solved non-symbolic small, exact addition problems compared to unknown addend problems. However, when children did successfully solve either kind of problem, they were able to use those derived solutions to solve a novel non-symbolic small, exact problem. These results suggest that the parallel individuation system is computationally flexible, contrasting with previous work showing that the AMS is more computationally limited, and shed light on the computational capacities and limitations of representing and operating over representations of small quantities of individual objects.

## INTRODUCTION

Humans are able to mentally represent the quantity of a set of objects in the world without the use of number words or digits. Research over the past several decades has suggested at least two formats for representing quantity in the mind that are available to humans: an analog magnitude system for representing the approximate numerosity of sets of larger quantities of objects (e.g., sets of greater than about four items), and a parallel individuation system for representing small quantities of individual 3-D objects (about four or fewer) in parallel (Dehaene, 1997; Feigenson et al., 2004; Odic & Starr, 2018; Wang & Kibbe, 2024). Both systems come online early in infancy (Coubart et al., 2014; Izard et al., 2009; Libertus & Brannon, 2009; Martin et al., 2022; Xu, 2003).

While both systems represent quantity, these representations have fundamentally different formats. The analog magnitude system allows humans to represent the quantity of a set of objects as a magnitude that roughly corresponds to the quantity of individual items in the set. Analog magnitude representations do not contain information about the individual objects in the set, but instead represent a summary of the quantity of those objects. Analog magnitude representations are imprecise (and get noisier as the number of objects in the set increases), but they represent the quantity of a set of objects explicitly: larger quantities are represented with larger magnitudes, smaller quantities with smaller magnitudes (Gallistel & Gelman, 1992, 2000; Halberda & Feigenson, 2008; Meck & Church, 1983). By contrast, the parallel individuation system represents each individual object in a small set by deploying discrete, index-like representations for each object, which act as attentional “pointers” to each object’s location (Feigenson & Carey, 2003, 2005; Leslie et al., 1998). The quantity of small sets of objects is therefore represented precisely, but only implicitly, via one-to-one correspondence between the object representations in the mind and the objects they represent in the world, and the quantities that this system can represent are limited (to set of objects with ∼4 or fewer objects; see Wang & Kibbe, 2024, for review).

Before children master formal symbolic mathematics, they can update both types of non-symbolic representations of quantity in response to real-world changes in quantity, performing what are commonly referred to as “arithmetic” operations over non-symbolic representations (Barth et al., 2006; Booth & Siegler, 2008; Cheng & Kibbe, 2023a; Christodoulou et al., 2017; Gilmore & Spelke, 2008; McCrink & Wynn, 2004; Wynn, 1992). Much of the research on non-symbolic “arithmetic” has focused on examining operations over AMS representations and how those operations are carried out. This research has shown that children can update analog magnitudes by incrementing or decrementing those representations by some magnitude. For example, Barth et al. (2006) showed 5-year-old children two large sets of dots that were hidden sequentially behind an occluder, removed the occluder to reveal a new set, and asked children whether the new set was larger or smaller than the total of the two sets together. To succeed, children needed to represent the initial quantity, update that representation to accommodate the second quantity, and then compare that updated representation to their representation of the revealed array. Children were able to approximately estimate the total quantity of the two sets together. Children also are able to perform operations over AMS representations that involve scaling those representations up or down by a factor of, e.g., 2 or 4 (McCrink et al., 2017; McCrink & Spelke, 2010, 2016; McCrink & Wynn, 2007), suggesting that AMS representations can be manipulated in a variety of computationally useful ways.

Furthermore, children can perform operations over AMS representations that require children to hold two representations in mind and perform an operation over those representations to derive a solution, in addition to being able to increment or decrement a single AMS representation. For example, Kibbe and Feigenson (2015, 2017) found that 4–6-year-old children could solve for unknown addends in non-symbolically-presented problems that used large quantities represented by the AMS. In their study, children were introduced to an animal character who had a “magic cup” which would add more objects to a visible set of objects. Children observed the set of objects before the animal added the set inside the cup, and then again after the contents were added, and were asked to select from two alternatives which quantity had been inside the cup. Children were able to select the correct quantity at rates above chance (effectively solving problems of the format, e.g., 5 + [addend] = 17; see also Cheng & Kibbe, 2023a). These results suggest that children can combine AMS representations using a computation that resembles solving for an unknown addend in symbolic math, in addition to manipulating a single representation (as in summation, subtraction, or scaling).

However, although a range of arithmetic-like operations can be performed over AMS representations, recent research suggests that the AMS may be computationally limited when it comes to being able to perform computations with the *solutions* to AMS operations. Specifically, children struggle in tasks that require them to use the solutions to AMS operations (i.e., solved addends) as inputs into new operations (i.e., adding the solved addend quantity to a new quantity). Cheng and Kibbe (2023b) first showed 4-8-year-old children two unknown-addend problems presented non-symbolically (as in Kibbe & Feigenson, 2015, 2017), and then showed them a new problem—children saw two unequal sets of objects and were asked to choose which of the two solved addends should be added to the smaller set of objects to make the sets “about the same”. To succeed, children needed to successfully solve for two unknown addends, and then select which of the two solutions should be used as an addend in a new, balancing operation, without ever having directly observed the quantities they were selecting from. Cheng and Kibbe (2023b) found that, while children could solve for both unknown addends with high fidelity, they were unable to use those solutions as inputs into a balancing operation. Children’s failure was not due to an inability to understand the balancing operation itself; children were successful when they were asked to perform a similar computation with visible quantities of objects rather than the (not directly observed) solutions to unknown addend problems. Cheng and Kibbe’s (2023b) results suggest an important limitation on the computational power of non-symbolic arithmetic over AMS representation: while children can solve arithmetic-like problems using the AMS, they were unable to use those solutions beyond the context in which the solutions arose. This means that AMS “arithmetic” does not have the combinatorial power of a true arithmetic (Dedekind & Beman, 1901).

While much research has examined the computational capacity of the AMS, the computational range of the parallel individuation system is less well understood. The distinct formats of AMS representations and individual object representations mean that the operations that are supported by these systems are executed in different ways. Research has shown that children can update individual object representations by updating the number of object indexes they have deployed to track objects in the set (Leslie et al., 1998). For example, Wynn (1992) showed infants scenarios in which a single object was placed on a puppet stage, the object was then occluded, and then a second object was added. In order to represent the quantity of objects that was hidden behind the occluder, infants needed to hold the first object index in mind, and then add a second index as the new object was added. Wynn (1992) found that infants were able to do so, as evidenced by their increased attention to the display when the occluder was lifted and the wrong quantity was revealed, suggesting that it is possible to carry out addition operations on individual object representations (for replications, see Berger et al., 2006; Clearfield & Westfahl, 2006; Cohen & Marks, 2002; Simon et al., 1995; Slater et al., 2010; Uller et al., 1999; Walden et al., 2007; see also Kibbe & Feigenson, 2017, for converging evidence from 4–6-year-olds). Similar results were obtained with subtraction operations in infants (Wynn, 1992).

However, one paper (Kibbe & Feigenson, 2017) found that 4–6-year-old children were unable to solve for an unknown addend when non-symbolic addend-unknown problems were instantiated using small quantities represented by individual object representations—an operation that is readily accomplished over AMS representations. For example, children who were shown problems like 1 + [addend] = 3 chose at chance when asked whether 1 or 2 objects had been added. By contrast, children had no difficulty when the problems were of the form 1 + 2 = [sum], readily selecting 3 as the correct answer over distractor quantities 2 or 4. These results suggest that some arithmetic operations may be more readily performed over individual object representations than others, and that the parallel individuation system may be more computationally limited than the AMS.

Together, the above studies suggest that addition and subtraction operations may be performed over individual object representations (i.e., by creating or removing object indexes in the face of real-world changes to object quantities; Kibbe & Feigenson, 2017; Wynn, 1992; see Leslie et al., 1998), but children may not be able to hold two sets of individual object representations in mind and compute their difference (i.e., represent a set of one individual object, and then a set of three individual objects, and compute that the difference between the sets is two; Kibbe & Feigenson, 2017). However, the source of the limitations on computations that can be performed over individual object representations is not entirely clear. It is possible that the representational format of individual object representations makes certain computations more accessible than others. For example, in summation or subtraction problems, children could deploy a new visual index or remove a visual index as the array quantities change (i.e., as objects are added or subtracted). Such visual updating may not be possible when children are asked to perform a computation over one or more *represented* sets, as in the unknown addend problems in Kibbe and Feigenson (2017) (see also Uller et al., 1999). It is also possible that, by virtue of the fact that children are required to manipulate multiple set representations, the increased working memory demands could make unknown-addend computations more error prone. If this is the case, children’s ability to use the parallel individuation system to compute small-quantity unknown addend problems should improve with age, as their working memory capacities increase substantially (Cheng & Kibbe, 2022; Simmering, 2012).

It is also not known whether children can perform additional computations *with the solutions* to non-symbolic arithmetic problems over small quantities, and whether this depends on the kind of arithmetic operation children are asked to perform. Previous work by Cheng and Kibbe (2023b) found that 4–6-year-olds could not reliably use the solutions to AMS arithmetic problems in a new AMS problem, suggesting a significant computational limit on the AMS that is not shared with formal, symbolic arithmetic, which is combinatorial by definition (Dedekind & Beman, 1901). However, whether the parallel individuation system would be subject to such a computational limit is an open question, and answering this question is important for several reasons. First, understanding whether *manipulated* representations of individual objects—that is, representations that arise from a computation in the *mind*, rather than strictly from an interaction with objects in the world—can be operated over in the mind is critical for our theoretical understanding of how individual object representations might be used in the mind beyond the context in which they arose. Second, since children typically start to learn formal arithmetic by manipulating small numbers, understanding the computational arithmetic capacity of the parallel individuation system has implications for the extent to which children can leverage their early, non-symbolic arithmetic systems to help them learn the formal rules of symbolic arithmetic.

Here, in two experiments, we examined children’s ability to perform arithmetic-like operations over small quantities and whether they can use the solutions to those computations as inputs into new non-symbolic, small-quantity problems. We asked children to compute two types of arithmetic-like problems instantiated with small quantities of objects (within participants): sums and unknown addends. We used the same age range as Kibbe and Feigenson (2015, 2017), 4–6-year-old children. Children of this age are learning counting and cardinality, but are not likely to yet have extensive experience with the symbolic forms of addition or unknown addend problems (National Governors Association Center for Best Practices, Council of Chief State School Officers, 2010). All children completed two blocks of trials, each of which required a different type of operation. In a Summation block, children solved for two summation problems presented non-symbolically. Children were told that two animal characters were collecting buttons inside opaque cups, and that they both liked to collect different numbers of buttons. Children then viewed three examples of each animal collecting buttons—one animal collected 1 + 2 buttons inside their cup, and the other collected 1 + 1 buttons inside their cup. In the critical Test trial, children were shown two new sets of buttons, one of which contained fewer than the other, and were asked to choose which of the two characters’ sums to add to the smaller set to make the sets have the same number of objects. To succeed, children needed to use the sums they computed, but never directly observed, as inputs into a balancing operation. In an Unknown Addend block, children solved for two unknown addends in problems presented non-symbolically. Children were shown two animal characters and were told that each animal has a “magic cup” that always adds a specific quantity to a set, but that we could not see what was inside the cup, we had to figure it out. Children then observed three examples of each character’s cup adding to a set and then revealing the set increased by some quantity (e.g., 1 + [cup] = 3; 1 + [cup] = 2). In the critical Test trial, children were shown two unequal sets and were asked to select which addend they should add to the smaller set to make the sets equal. To succeed, children needed to use the solved addends that they computed, but did not directly observe, as inputs in a balancing operation.

We had two primary goals for these experiments. First, we aimed to replicate and extend Kibbe and Feigenson’s (2017) result that children can successfully solve non-symbolic small-quantity sums but have more difficulty with small-quantity unknown addends. We added several checks beyond what Kibbe and Feigenson (2017) did to examine children’s representations of the solutions to non-symbolic small-quantity sums and unknown addends, and to determine whether we can observe variability in children’s ability to solve for each type of problem. Our within-participants design enabled us to examine whether children’s ability to solve small-quantity non-symbolic summation problems is correlated with their ability to solve small-quantity, non-symbolic unknown-addend problems. We also tested a larger sample of children, sufficiently powered to be able to detect any effects of age on children’s performance. Thus, we aimed to better characterize children’s ability to perform computations over individual object representations during a period of time in which children’s working memory capacities are increasing substantially.

Our second goal was to investigate whether the outputs of computations over small quantities of objects can be used as inputs into new computations. That is, we asked whether we would observe the same context-dependent computational limitation that has previously been observed in the AMS when children are performing operations over individual object representations.

## EXPERIMENT 1

### Methods

#### Participants.

Forty-nine 4- to 7-year-olds (mean age = 5.86 years, age range: 4 year 0 months 2 days–7 years 0 months 14 days, 26 girls) participated in Experiment 1. The study was conducted remotely via Zoom. This sample size was similar to a study with a similar design that examined the development of 4–6-year-old children’s AMS computational capacity (Cheng & Kibbe, 2023b), in which age-related changes in capacity were observed. Two additional children participated but were excluded from analyses because they declined to complete all study procedures.

Participants were recruited from the greater Boston area through public birth records, family events, and social media. Thirty out of 49 families answered the optional demographic form. Parents reported their child to be Asian (4), Asian African (1), Asian White (4), American Indian (1), White (19), or other (1). One family reported their child to be Hispanic or Latinx, 28 families reported their child to be not Hispanic or Latinx, and one family preferred not to say. For all children, at least one caregiver reported having a college degree or higher. Each family received a $10 Amazon gift card for their participation in the study. The study was approved by the Boston University Charles River Campus Institutional Research Board under protocol number 3618E.

#### Apparatus and Stimuli.

Families were asked to participate in a quiet room from home. The experimental stimuli were created in Keynote Presentation software (full stimuli, plus a video demonstrating an example experimental session, are available at https://osf.io/jkc5f/). The stimuli included images of buttons of varying sizes presented on a computer or tablet screen (on a 13.3 inch laptop, the buttons’ diameters ranged from .5 cm to .7 cm). The button images used were similar across the study blocks, except for their color (orange in the Summation block, blue in the Unknown-addend block). During the experiment, the experimenter shared their screen using the Zoom screen-share function to enable children to view the stimuli. Families were asked to participate using a device that had a screen 10 inches or larger (43 families used a laptop, 3 families used a desktop computer, and 3 families used a tablet). The experimenter recorded the experimental session with the permission of the caregivers.

#### Procedure.

##### Screen Set-Up.

The experimenter first guided caregivers through the Zoom set-up for the experiment. Caregivers were instructed on how to hide self-view and place the experimenter’s video in the top center of the screen. The experimenter then shared their screen so that children could view the stimuli controlled by the experimenter on her computer, and checked with caregivers that children could see the stimuli and hear the experimenter clearly.

Children then completed a series of Pre-trials followed by two blocks of computation trials: a Summation block and an Unknown Addend block (block order counterbalanced across participants).

##### Pre-Trials.

###### Balancing Familiarization.

**Figure 1.**
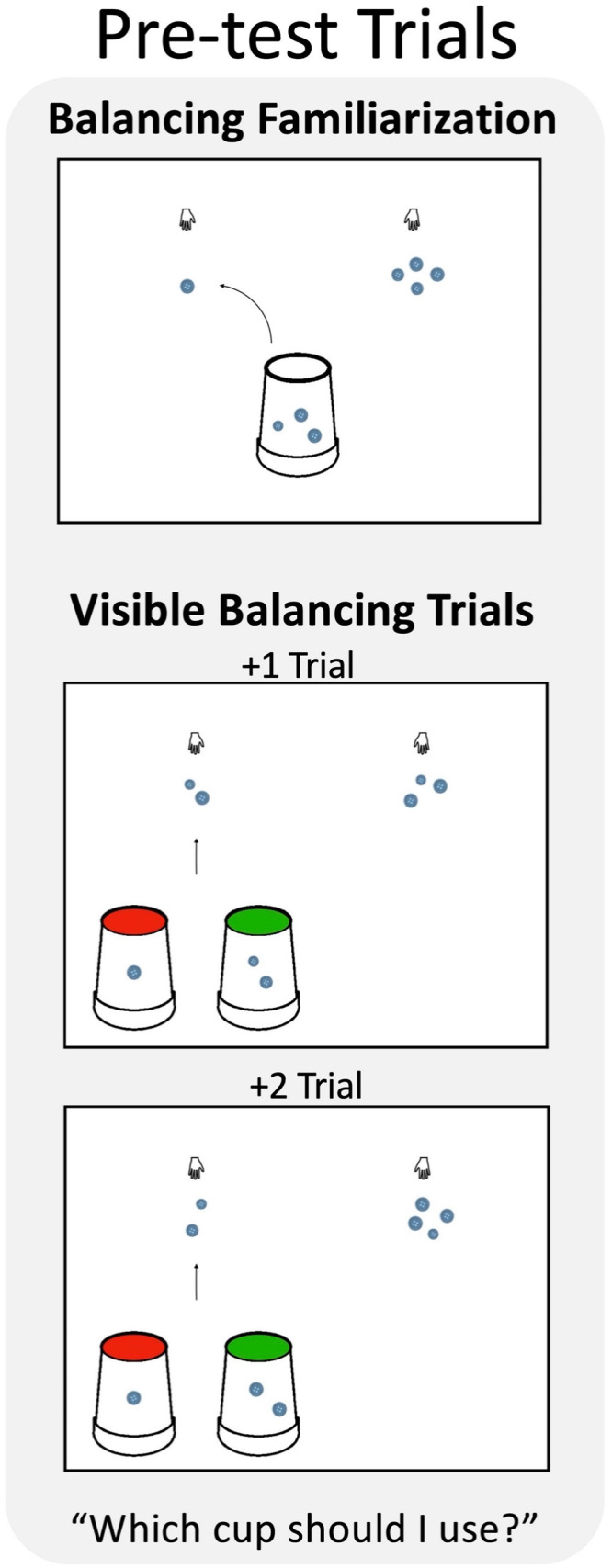
Pre-test trials from Experiment 1. Children completed a Balancing Familiarization trial, and then two Visible Balancing trials in which they were asked to select which of two visible quantities should be added to the smaller set to make the two sets the same.

To orient children to the action of balancing unequal sets of objects by adding objects to the smaller of the two sets, we first had children complete a Balancing Familiarization trial (Figure 1). The experimenter showed two unequal sets of buttons (a set of one and a set of four buttons) and said, “See these two piles of buttons? They have different numbers of buttons. But I want them to be the same.” Then a transparent cup containing three buttons appeared on the bottom center of the screen, and the experimenter said, “I have a magic cup!” An arrow appeared above the cup pointing to the smaller set, and the experimenter continued, “I want to use my magic cup to add to this pile [arrow indicating the smaller set], so that these two piles will look the same.” Then the experimenter played an animation in which the cup moved to cover the smaller set, added the buttons to the set, and then moved out of the display. After that, the experimenter showed children which buttons were added by playing an animation that caused the added buttons to flash a different color, and the experimenter said, “See the flashing ones? Those are the buttons that the cup just added. Now these two piles have the same number of buttons!”

###### Visible Balancing Trials.

To obtain a baseline measure of children’s ability to balance unequal sets, children completed two Visible Balancing trials (Figure 1). On each trial, the experimenter showed children two unequal sets of buttons (in one trial: a set of 2 and a set of 3; in the other trial: a set of 2 and a set of 4; trial order counterbalanced across participants) and told children that she wants them “to be the same”. The experimenter then advanced the animation to present two transparent cups, a red-outlined cup containing one button and a green-outlined cup containing two buttons. The experimenter then said, “This time I have two cups. Which cup should I use to add to this one [an arrow appeared pointing to the smaller set] so that these two piles will look the same? The red cup, or the green cup?” After children answered, the experimenter gave them feedback by moving their selected cup over the smaller set and adding the buttons. If children were correct, the experimenter encouraged children to observe the two sets and said, “Yes, right! Good job!” Then the experimenter animated the added buttons so that they flashed a different color, and continued, “See the flashing button(s)? This is the one [these are the ones] that my [red/green] cup just added! Now these two piles have the same number of buttons!” If children were incorrect, the experimenter said, “Now do you think these two piles have the same number of buttons?” If children said no, then the experimenter proceeded, “No, right? Ok, so what do you think, shall we try the other cup?” If children still had difficulty, the experimenter prompted children to count with her and then encouraged children to try the other cup. On the trial with sets of two and three buttons, the red cup (containing one button) was the correct answer (+1 trial), and on the trial with sets of two and four buttons, the green cup (containing two buttons) was the correct answer (+2 trial; see Figure 1).

Children then proceeded to complete two blocks of computation trials: A Summation block and an Unknown Addend block. The order of the blocks was counterbalanced across children. Below, we describe the Summation block first, followed by the Unknown Addend block. Each block included four phases: Familiarization, Demonstration, Test, and Post-test.

##### Summation Block.

###### Familiarization Trial.

The experimenter first familiarized children with the summation animation. An orange cup appeared on the lower center of the screen. The experimenter said, “This is my orange cup. I’m going to put some buttons in the cup, and I want you to help me figure out how many buttons my orange cup is going to collect. Now watch this.” A first set of buttons (two) appeared on the left side of the screen, and the experimenter continued, “Here are some buttons, I’m putting them in my cup.” The experimenter then advanced the animation so that the cup moved to cover the set. Then, a second set of buttons (three) appeared on the right side of the screen. The experimenter said, “Here are some more buttons. I’m putting them in my cup too.” Then the cup moved to cover the second pile. The cup then moved back to its original location. The experimenter then showed children how many buttons the cup had collected by turning the cup transparent to reveal a total of five buttons inside the cup, and said, “See the buttons we collected?”

###### Demonstration Trials.

**Figure 2.**
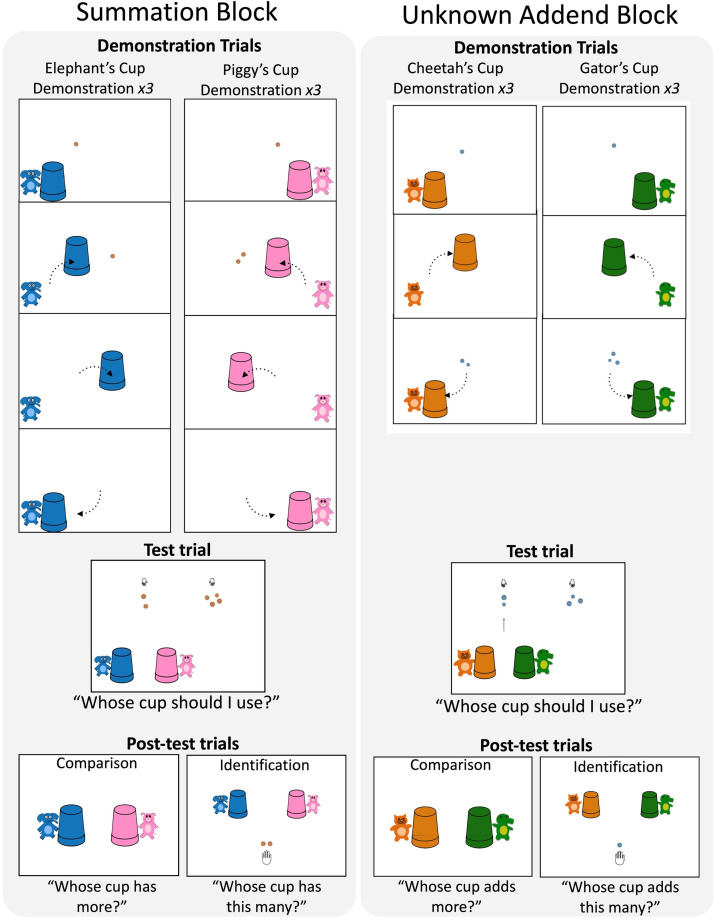
Example Demonstration, Test, and Post-test trials from the Summation block and Unknown Addend blocks of Experiment 1. Block order was counterbalanced across participants.

Children next completed two sets of Demonstration trials (see Figure 2, left panel). First, the experimenter showed children images of an elephant character with a blue cup on the bottom left of the screen, and a pig character with a pink cup on the bottom right of the screen (loosely styled after Gerald and Piggy from the *Elephant and Piggy* books by Mo Willems). The experimenter said, “Now I want to introduce my friends. This is Elephant [the elephant bounced] and Elephant’s cup [the cup jiggled]. This is Piggy [the pig bounced] and Piggy’s cup [the cup jiggled].” The experimenter further explained, “They each like to collect different numbers of buttons in their cups. Elephant has a favorite number of buttons he likes to collect, and Piggie has a favorite number of buttons she likes to collect. But I don’t know how many they each like best. Can you help me figure it out? Here’s how we are going to figure it out. We are going to add some buttons to their cups, just like I did with my orange cup, and they will let us know when they have just the right number of buttons in their cups. But this time, we won’t get to see the buttons inside the cup, so we have to figure it out ourselves. Are you ready?”

Children then observed three Demonstration trials for each cup, following a similar method used by Kibbe and Feigenson (2017) (three Elephant demonstrations and three Piggy demonstrations; whether children observed the Elephant demonstrations or the Piggy demonstrations first was counterbalanced across children). Here we describe the condition in which Elephant trials were presented first, followed by Piggy trials.

At the start of the Elephant trials, children saw a screen depicting only Elephant and his blue cup in the bottom left. The experimenter said, “Here’s Elephant and his cup. Elephant is going to collect some buttons. Let’s see how many he’s going to collect!” In the first Elephant trial, a single button appeared on the left side of the screen, and the experimenter said, “See this button? Let’s put it inside Elephant’s cup.” The cup then moved to cover the button. Another single button then appeared on the right side of the screen, and the experimenter again said “See this button? Let’s put it inside Elephant’s cup.” The cup moved to cover the second button. After the second button was added to the cup, Elephant was animated to jump up and down, and the experimenter said, “Hooray! We gave Elephant just the right number of buttons!” The cup then moved back to its original location next to Elephant. The second and third Elephant trials proceeded similarly, except at the beginning of each trial, the cup turned transparent to show children that it starts each trial empty inside (“Let’s try again. See Elephant’s cup is empty?”) The cup then turned opaque again, and the experimenter continued with the trial. At the end of each Elephant trial, Elephant’s cup always contained two buttons.

At the start of the Piggy trials, children saw a screen depicting only Piggy and her pink cup in the bottom right. The experimenter said, “This is Piggy and her cup. Piggy’s favorite number is different than Elephant’s. Piggy’s cup collects a *different number* of buttons than Elephant’s cup does. Can you help me figure out how many buttons does Piggy collect?” On each Piggy trial, children observed Piggy’s cup collect a set of one button and a set of two buttons, ending up with three total (whether the cup collected one or two first was counterbalanced across the three trials: Children saw 1 + 2, 2 + 1, and 1 + 2). We used the same sizes of buttons across the three Demonstration trials.

###### Test Trial.

In the single Test trial (Figure 2, left panel), we asked whether children could use their solutions to the problems in the Demonstration trials as inputs into a balancing operation. The experimenter showed children two sets of buttons and said, “See these two piles of buttons? They have different numbers of buttons. But I want them to be the same.” Then the two characters, Elephant and Piggy, and their cups, appeared under the smaller set. The two characters bounced while the experimenter continued, “Whose cup should I use to add more buttons to *this* one [an arrow pointed to the smaller set] so that these two piles will look the same?” Children did not receive feedback during the Test trial. Half of the children completed Test trials in which Elephant’s cup was the correct answer (+Elephant trial), while for the other half of children, Piggy’s cup was correct (+Piggy trial; see Table 1).

**Table 1.** Description of the quantities used in the Demonstration and Test trials and summary of children’s responses on Test and Post-Test trials in Experiments 1 and 2. *p* Values represent the results of statistical comparisons against chance (50%).

	Demonstration Quantities (three trials)	Test Quantities	Test Response	Post-Test Response
Exp. 1	*Summation*: 1 + 1 = Elephant; 2 + 1 = Piggie	+Elephant (2): 2 vs. 4 or +Piggie (3): 2 vs. 5	80%, *p* < .001	Comparison: 86%, *p* < .001
				Identification: 90%, *p* < .001
	*Unknown-addend*: 1 + Gator = 3; 1 + Cheetah = 2	+Gator (2): 2 vs. 4 or +Cheetah (1): 2 vs. 3	65%, *p* = .044	Comparison: 81%, *p* < .001
				Identification: 71%, *p* = .004
Exp. 2	*Summation*: 1 + 1 = Elephant; 2 + 1 = Piggie	+Elephant (2): 2 vs. 4 or +Piggie (3): 2 vs. 5	80%, *p* < .001	Comparison: 78%, *p* < .001
				Identification: 78%, *p* < .001
	*Unknown-addend*: 1 + Gator = 3; 2 + Cheetah = 3	+Gator (2): 3 vs. 5 or +Cheetah (1): 3 vs. 4	50%, *p* = 1	Comparison: 56%, *p* = .48
				Identification: 55%, *p* = .67

###### Post-Test Trials.

Children then completed two Post-test trials (Figure 2, left panel), which were designed to directly measure children’s representations of the quantities in each cup (i.e., to test whether they had solved the summation problems in the Demonstration trials). In each trial, children saw a screen with Elephant and Piggy, each paired with their cups. In the Comparison trial, the experimenter asked, “Whose cup has more buttons?” In the Identification trial, the experimenter advanced the animation so that either two or three buttons (counterbalanced across children) were visible in the lower center of the screen. The experimenter then asked, “Whose cup has this many?” Children did not receive feedback during the Post-test trials.

##### Unknown Addend Block.

###### Unknown Addend Familiarization.

In the Unknown Addend block, to orient children to the unknown-addend format of the non-symbolic problems, children first observed a single familiarization trial (for a similar approach, see Cheng & Kibbe, 2023b). In the first trial, children saw an opaque blue cup on the bottom center of the screen. The experimenter said, “This is my blue cup [the cup jiggled]. There are some buttons inside my cup. But I don’t know how many buttons are inside my cup. Want to help me figure it out?” A single button appeared then on the top center of the screen, “OK watch this, here is a button. My cup is going to come and add some more buttons. We can look at the buttons before my cup adds, and the after my cup adds, and figure out how many buttons were in my cup. Ready?” The cup then moved to cover the button and then moved back to its original location, revealing four buttons on the top center of the screen. The experimenter then said, “Ok, let’s look at the buttons now! Can we figure out how many my cup just added? Now watch this.” The experimenter animated the display so that the added buttons flashed a different color, and said, “See the flashed buttons? Those are the ones that my cup just added. So now we know how many buttons were in my cup! We looked at the buttons before my cup was added, and after my cup was added, and figured out how many were in my cup.”

###### Demonstration Trials.

Demonstration trials proceeded similarly to the Summation block (see Figure 2, right panel), following a similar method to Kibbe and Feigenson (2017). The experimenter introduced Cheetah and his orange cup and Gator and his green cup. The experimenter said, “They have different numbers of buttons in their cups. But I don’t know how many they have in each of their cups. Can you help me figure it out? Here’s how we are going to figure it out. We are going to use Cheetah’s and Gator’s cups to add to some piles of buttons. We’ll look at the piles of buttons before each cup adds, and the buttons after each cup adds, and we’ll try to figure out how many each cup added. But we won’t get to see the buttons flashing this time. We have to figure it out ourselves. Are you ready?”

Children then saw two sets of Demonstration trials, one set for Cheetah’s cup and one set for Gator’s cup, each consisting of three trials (whether Cheetah’s or Gator’s demonstrations were presented first was counterbalanced across children). At the start of the Cheetah set of demonstrations, the experimenter showed a screen in which Cheetah and an orange cup were visible in the lower left corner. The experimenter played an animation to jiggle the cup and said, “Here’s Cheetah and his cup. Cheetah’s cup will add to a pile, but I don’t know how many it adds.” A set of one button then appeared in the center of the screen, “See this button? Let’s use Cheetah’s cup to add to this pile. Like this.” Cheetah’s cup then moved to cover the set of two buttons, and then moved back to its original location, revealing a final set of three buttons in the center. The experimenter then said, “Ok, see the buttons now?” Children then saw two more trials in which Cheetah’s cup added to a set of one, yielding a final set of two buttons.

The experimenter then showed children Gator and the green cup (presented on the lower right side of the screen; Figure 2, right panel). The experimenter said, “This is Gator and his cup.” The cup jiggled while the experimenter said, “His cup is different than Cheetah’s, it adds a different number of buttons than Cheetah’s cup does. But I don’t know how many it adds.” The experimenter then showed children a set of one button in the center of the screen and said, “See this button? Let’s use Gator’s cup to add to this pile. Like this.” The green cup then moved to cover the center set and then moved back to its original location, revealing a final set of three buttons in the center. The experimenter then said, “See the buttons now?” The next two trials proceeded similarly, with Gator’s cup adding to a set of one button and yielding a final set of three buttons.

We used the same set of different sized buttons across trials. Within each trial, children could potentially identify which objects were part of the initial set after the cup adds (i.e., they could use the sizes of the objects to re-identify the original set among the added objects).

###### Test Trial.

The single Test trial examined whether children could use the solutions to the unknown-addend problems in the Demonstration trials to perform a balancing operation. The Test trial proceeded similarly to the Test trial in the Summation block (see Figure 2, right panel). Children were shown two unequal sets of buttons, a set of two and a set of either three or four, and were asked to select which character’s cup should be added to the smaller set to make the sets the same. Half of the children completed a Test trial in which Cheetah’s cup was the correct choice (+Cheetah trial); for the other half of children, Gator’s cup was the correct choice (+Gator trial; see Table 1).

###### Post-Test Trials.

Post-test trials proceeded similarly to the Summation block, except that the prompt questions were worded to reflect the fact that children were being asked about the *addend* rather than the final total (see Figure 2, right panel). In the Comparison trial, children were asked, “Which cup adds more?” and in the Identification trial children were asked “Which cup adds this many?”

###### Quantities.

We attempted to select quantities that were representable using individual object representations while also reducing repetition of quantities between the Demonstration and Test trials. Since the small number range limits such quantity options, we made the decision to include sets of five objects as the “larger” of the two uneven sets in some of the Test trials (in both Experiment 1 and Experiment 2; see Table 1). Previous work has shown that sets of five are border cases for non-symbolic representation; they can be represented using exact, non-symbolic representations and can be compared via one-to-one correspondence, but children have more difficulty tracking these sets when the elements in the set are manipulated (Izard et al., 2014). Since sets of five can be represented exactly, and since these sets were presented as static arrays in our experiments (i.e., arrays that children had to compare to the smaller set in the Test trial via one-to-one correspondence), we decided to include these quantities. To examine whether including the set of five impacted children’s performance, we conducted analyses comparing children’s performance between Test trials containing the set of five with Test trials containing a smaller set for the larger quantity, which are presented in the Results sections for each experiment. Quantities 1–4 are represented exactly in children in our age range (Hutchison et al., 2020).

### Results

#### Visible Balancing Trials.

Children’s proportion correct across the two Visible Balancing trials was .82, significantly greater than would be expected if children were choosing randomly (chance = .5; Wilcoxon sign rank test *p* < .001). Children’s proportion correct responses were not significantly correlated with their age in years (*r* = .27; *p* = .058), although older children tended to have more success. These results suggest that children in our sample were able to perform a balancing operation over visible sets of small numbers of objects.

#### Test Trial.

**Figure 3.**
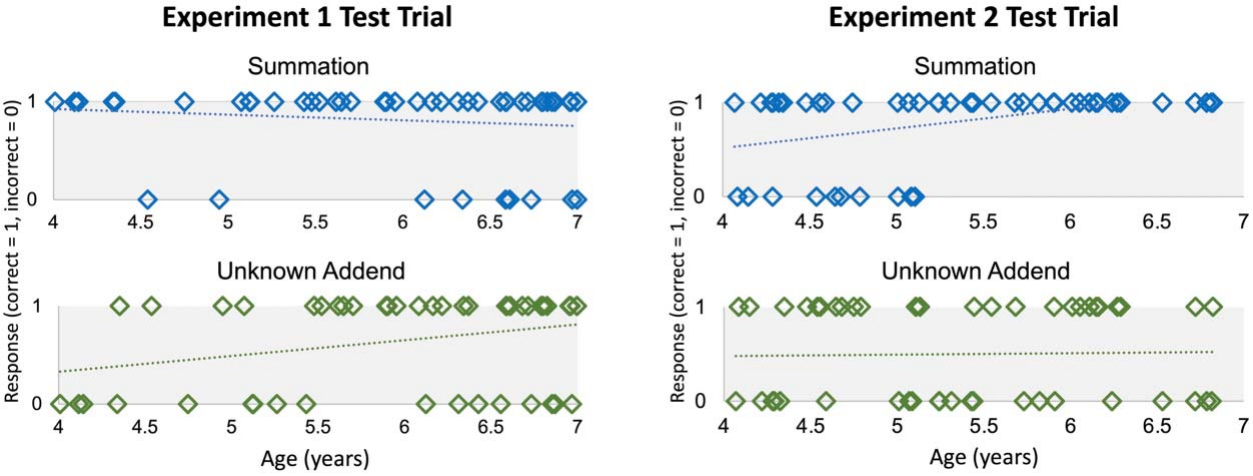
Individual children’s responses (correct or incorrect) in the Test trials of Experiments 1 and 2.

Children’s responses in the Summation and Unknown Addend Test trials are shown in Figure 3 and summarized in Table 1. In the Summation block, 39/49 children (80%) correctly selected the cup containing the sum that, when added to the smaller set, would balance the two sets (chance = 50% binomial test *p* < .001), and children performed similarly whether they completed the +Elephant Test trial (20/25, 80%) or the +Piggy Test trial (19/24, 79%) (Fisher’s exact test *p* = 1), suggesting there was no difference in children’s performance between trials in which the larger set contained five objects and trials in which the larger set contained four. In the Unknown-addend block, 32/49 children (65%) correctly selected the solved addend that would balance the two sets (binomial test *p* = .044, chance = 50%); children who had the +Gator Test trial did slightly worse (14/25 correct, 58%) compared to children who had the +Cheetah Test trial (18/25 correct, 72%), but this difference was not statistically significant (Fisher’s exact test *p* = .377). We observed no effects of block order or demonstration trial order (*p*s > .25).

While children made overall fewer correct responses in the Unknown Addend Test trial compared to the Summation Test trial, this difference was not statistically significant (McNemar Test = 1.71, *p* = .189). Children’s performance on the Summation Test trial did not significantly correlate with their performance on the Unknown Addend Test trial (*r* = −.05, *p* = .558). Age was (weakly) correlated with Unknown Addend Test trial responses (*r* = .28, *p* = .049), but not with Summation Test trial responses (*r* = −.09, *p* = .558; see Figure 3).

#### Post-Test Trials.

To better understand the representations driving children’s performance in the Test trial, we examined children’s responses in the Post-test trials, in which we directly measured the precision of children’s representations of the quantities in the cups. In the Comparison trial, children selected the cup with the greater number in both the Summation (42/49 children, 86%) and the Unknown-Addend (40/49 children, 82%) blocks (chance = 50%; binomial test, both *p* < .001), and their accuracy did not differ significantly between the two blocks (McNemar’s Test = .08, *asymp. p* = .773). In the Identification trial, children identified the correct target cup in both the Summation (44/49 children, 90%) and Unknown Addend blocks (34/48 children, 71%; one child declined to respond on this trial) at rates above chance (binomial test, both *p* < .004). However, children were significantly better at identifying the target cup when the quantity was the result of summation compared to the result of an unknown-addend operation (McNemar’s Test = 4.27, *asymp. p* = .039). No age-related differences were observed in either the Comparison or the Identification trial in either block (all *p*s > .056, *r*s < .277). Together, these results suggest that, while children were fairly certain about which cup contained the greater number of objects in both the Summation and Unknown Addend blocks, their representations were more precise in the Summation block.

### Discussion

First, we found that children were able to solve for two sums, and then use those sums as inputs into a balancing operation, selecting which of the two sums should be added to the smaller of two sets to make the sets “the same”. To answer correctly, children needed to compute two sums across sets of Demonstration trials, maintain bindings between each sum and a specific character’s cup, and then deploy the computed sum to solve a new problem. Crucially, children never directly observed the sums; instead, they observed two addends and had to compute their sum. Children’s success in the Summation Test trial—and their accuracy in the post-test trials designed to examine the precision of children’s representations—suggests that children can compute sums and work directly with those solutions in non-symbolic problem contexts.

Second, we aimed to examine whether children also could do so when the problems were in a more challenging unknown-addend format. We found that children showed evidence of being able to select which of two solved addends should be used to balance unequal sets, and their performance on the post-test trials suggested that they had solved for the unknown addends successfully. However, while they selected the correct unknown addend to balance the unequal sets in the Test trial at rates above chance, a binomial test only just reached statistical significance, and children made more errors when asked directly about the quantity of the solutions to unknown addend problems compared to summation problems. This suggests that children may have had more difficulty computing the solutions to non-symbolic unknown-addend problems, and this may be true across the age range we tested. Since both problems were presented with small quantities, and both problems involved similar quantities, the differences in accuracy between the summation and unknown addend solutions suggests that differences in performance across these two problem types is due to the different computational demands these problems make.

The fact that children showed some success at solving non-symbolic unknown-addend problems in Experiment 1 contrasts somewhat with the conclusions of Kibbe and Feigenson (2017) and suggests that children may have more competence with these kinds of problems than previously thought. However, it is possible that children could have succeeded using a lower-level strategy that did not require them to directly solve for unknown addends or compute with those solutions. Specifically, between the two unknown addend scenarios (Gator’s cup and Cheetah’s cup), the initial quantity (before the cup was added) was the same (1) while the quantity that was added by the cups differed (1 or 2). This meant that the final quantity after the cup was added was always greater when the *addend* was greater. That is, Gator’s final quantity was always larger than Cheetah’s. Some children may have used a strategy of attending to the final quantities, rather than solving directly for the unknown addend, and using the final quantities as a basis for their choices in the Test trial and for their judgments in the post-test trials (see also Cheng & Kibbe, 2023b). This could potentially explain why children were a) slightly (but not significantly) more successful in the Test trial in the Summation block, in which such a strategy was not possible, b) why children performed slightly (but not significantly) worse in the +Gator condition (some children may have selected Cheetah because his final quantity would balance the two sets), or c) why children were more successful in the Identification post-test trials in the Summation block compared to the Unknown Addend block.

We addressed this possibility in Experiment 2. Experiment 2 was nearly identical to Experiment 1, except that we made two changes to the quantities in the Unknown Addend block. First, we changed the *initial quantity* in the Unknown Addend Demonstration trials—while Cheetah’s cup always added one object and Gator’s cup always added two objects (as in Experiment 1), we varied the initial quantity so that the final quantity after the cups were added was always three. In this way, Gator and Cheetah always ended up with the same quantity after their cups were added (see Table 1), which meant that children could not rely on a strategy in which they attended only to the final quantities in the Demonstration trials to make decisions in the Test or Post-test trials. Second, we changed the quantities of the two unequal sets in the Unknown Addend block Test trial to 3 vs. 4 (+Cheetah condition) and 3 vs. 5 (+Gator condition), rather than 2 vs. 3 and 2 vs. 4, to ensure children were not carrying over the visible quantities from the Demonstration trials as cues to respond at Test. The Summation block was identical to Experiment 1, and thus served as a direct replication of the Summation block of Experiment 1.

## EXPERIMENT 2

### Methods

#### Participants.

Fifty children (mean age = 5.36 years, age range: 4 year 0 months–6 years 9 months, 31 girls) participated in Experiment 2 via Zoom. Sample size was determined as in Experiment 1. Two additional children were tested but were not included in analyses due to experimenter error (1) and declining to complete study procedures (1).

Forty-two caregivers answered the optional demographic form. Caregivers reported their child as Asian (9), Asian White (6), American Indian (1), White (24), or other (2). Two caregivers reported their child as Hispanic or Latinx, 37 reported their child as not Hispanic or Latinx, and three caregivers selected “prefer not to say”. Each child had at least one caregiver who was reported to have a college degree. The study was approved by the Boston University Charles River Campus Institutional Review Board (protocol #3618E).

#### Apparatus and Stimuli.

The stimuli, including the button images, cups, and animals, were similar to Experiment 1. All families participated using a laptop computer. The full stimuli and an example video can be found at https://osf.io/jkc5f/.

#### Procedure.

Experiment 2 followed the same procedures as Experiment 1, with two exceptions in the Unknown Addend block. First, we changed the quantity of the *first* set presented in each Unknown Addend Demonstration trial, so that the *final* quantities (after Gator’s or Cheetah’s cups were added) were the same (always 3; see Table 1). Specifically, to demonstrate Gator’s cup, the experimenter showed children a set of one button, showed Gator’s cup occluding the set, and then revealed the final set of three buttons. To demonstrate Cheetah’s cup, the experimenter showed children a set of two buttons, showed Cheetah’s cup occluding the set, and then revealed the final set of three buttons.

We also changed the quantities of the two unequal sets presented in the Unknown Addend Test trial (see Table 1). In the Test trial, children either saw a set of three and a set of four buttons (+Cheetah trial) or a set of three and a set of five buttons (+Gator trial).

### Results

#### Visible Balancing Trials.

Children’s proportion correct across the two Visible Balancing trials was .82 (chance = .5, Wilcoxon Signed Rank test *asymp. p* < .001). As in Experiment 1, although children’s proportion correct tended to increase with age, a correlation between proportion correct and age did not reach statistical significance (*r* = .268, *p* = .06).

#### Test Trial.

Children’s responses in the Summation and Unknown Addend block Test trials are shown in Figure 3 and summarized in Table 1. In the Summation block, 40/50 children (80%) selected the correct sum that would balance the unequal sets (chance = 50%; binomial *p* < .001), and children performed similarly whether they completed the +Elephant Test trial (19/25 children (76%) chose correctly) or the +Piggy Test trial (20/25 children (80%) chose correctly) (Fisher’s exact test *p* = 1); children performed similarly when the larger set contained five objects compared to when the larger set contained four objects. In the Unknown-addend Test trial, 25/50 children (50%) correctly chose the addend that would balance the unequal sets, not different from chance (binomial *p* = 1). Children responded similarly regardless of whether they completed the +Gator Test trial (14/26 children (54%) chose correctly) or the +Cheetah Test trial (11/24 children (46%) chose correctly) (Fisher’s exact test *p* = .78), suggesting that children’s performance was not significantly impacted by the inclusion of the set of five objects in the +Gator condition, compared to the relatively smaller quantities used in the +Cheetah condition. There were no effects of block order or demonstration trial order (*p*s > .72).

Children performed significantly better in the Summation block Test trial compared to the Unknown Addend block Test trial (McNemar test = 6.76, *asymp. p* = .009). Summation block Test trial performance was significantly correlated with age (*r* = .43, *p* = .002), but Unknown Addend block Test trial performance was not (*r* = .026, *p* = .860) (see Figure 3).

#### Post-Test Trials.

In the Comparison trial, children successfully selected the cup that contained the greater quantity in the Summation block (39/50 children (78%) responded correctly, binomial *p* < .001), but not in the Unknown Addend block (28/50 children (56%) responded correctly, binomial *p* = .48), and their correct responses were significantly different across the two blocks (McNemar test = 5.88, *asymp. p* = .015). In the Identification trial, children identified the correct target cup in the Summation block (39/50 children (78%) chose correctly binomial *p* < .001), but not in the Unknown Addend block (27/50 children (54%) chose correctly, binomial *p* = .67); children performed significantly worse in the Unknown Addend block compared to the Summation block (McNemar test = 4.76, *asymp. p* = .029). No age-related differences were observed in either the Comparison or the Identification trial in either blocks (all *p*s > .077, *r*s < .255). These results suggest that children successfully solved the sums in the Summation block, but they were not overall successful in solving for the unknown addends in the Unknown Addend block.

#### Experiment 1 and 2 Compared.

##### Visible Balancing Trials.

There were no differences in mean performance in Visible Balancing trials across the two experiments (independent samples *t* test *t*(97) = .056, *p* = .96). Age was significantly correlated with children’s performance in Visible Balancing trials (*r* = .23, *p* = .022), controlling for experiment; with increasing age, children were more likely to choose the correct set in Visible Balancing trials.

##### Test Trials.

Children performed similarly in the Summation Test trial of Experiments 1 and 2 (Mann-Whitney U test, Z = .05, *asymp. p* = .96). In the Unknown Addend Test trial, despite the fact that children chose the correct cup at Test at rates greater than chance in Experiment 1 (65%) and at rates not different from chance in Experiment 2 (50%), there was no statistically significant difference in children’s choices between the two experiments (Mann-Whitney U test, Z = 1.53, *asymp. p* = 125). For both blocks, children’s responses in the Visible Balancing trials were correlated with the Test trial responses controlling for experiment (Summation Block: *r* = .224, *p* = .027; Unknown Addend Block: *r* = .282, *p* = .005), suggesting that children’s ability to understand and perform a balancing operation may have supported their ability to compute with the solutions to the non-symbolic arithmetic problems. Across both experiments, children performed significantly better in the Summation Test trial than the Unknown Addend Test trial (McNemar test *χ*^2^ = 8.82, *asymp. p* = .003).

##### Post-Test Trials.

Children in Experiments 1 and 2 performed similarly in the Summation block Comparison (Z = .99, *asymp. p* = .32) and Identification (Z = 1.59, *asymp. p* = .11) trials. In the Unknown Addend blocks, children performed significantly better on the Comparison trial in Experiment 1 compared to Experiment 2 (Mann-Whitney U test, Z = 2.74, *asymp. p* = .006) but performed similarly in the Identification trial (Z = 1.56, *asymp. p* = .11), suggesting that children in Experiment 1 may have been basing their response in the Comparison trials on the final quantity revealed in the Demonstration trials rather than solving for the unknown addends directly. Overall, children performed significantly better in the Summation Post-Test trials compared to the Unknown Addend Post-Test trials (Comparison: McNemar test, *χ*^2^ = 4.97, *asymp. p* = .026; Identification: *χ*^2^ = 10.03, *asymp. p* = .002).

##### Relationship Between Test Trial Performance and Representational Precision.

**Figure 4.**
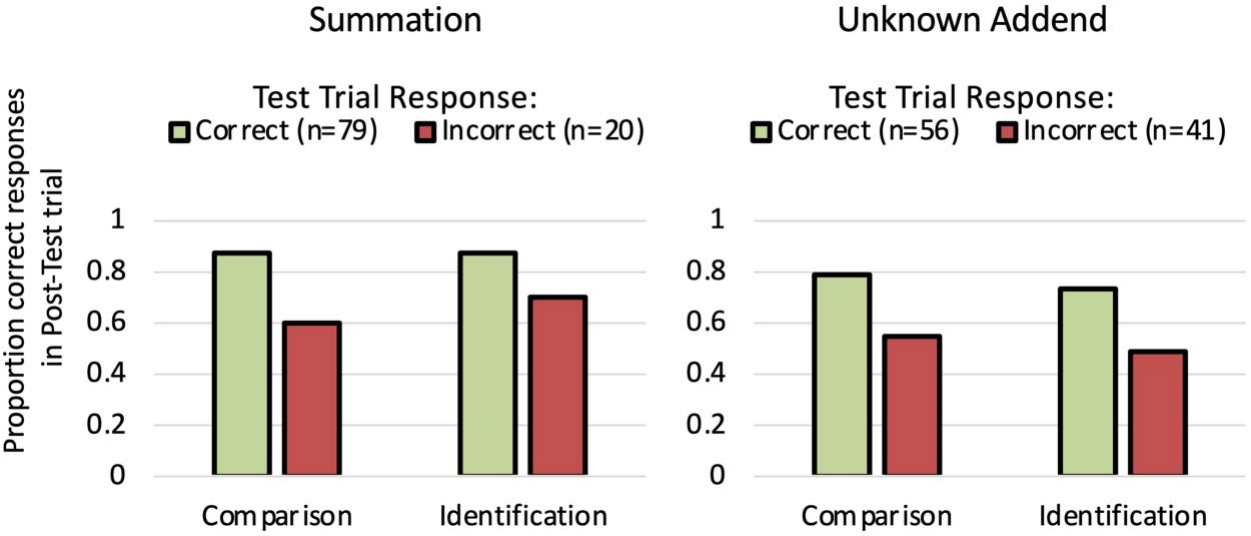
Children’s proportion correct responses in the Comparison and Identification trials of Experiments 1 and 2 (combined) as a function of whether they successfully chose which of the two solutions should be used to balance the unequal sets in the Summation Test trial (left panel) and the Unknown Addend Test trial (right panel).

Finally, we took advantage of our combined samples of Experiments 1 and 2 to ask whether there were any systematic relationships between children’s success in the Test trials, in which they were asked to select which of the two solutions (sums in the Summation block; solved unknowns in the Unknown Addend block) should be used to balance unequal sets, and their performance in the Post-Test trials, in which we measured the precision of those solutions. We divided children into two groups within each block—those who chose correctly in the Test trial and those who chose incorrectly—and asked whether children’s overall performance in the relevant Post-Test trial varied depending on their Test trial performance. These results are shown in Figure 4.

Children who responded correctly in the Summation Test trial (*n* = 79) were more accurate in the Summation Comparison trial than children who responded incorrectly in the Summation Test trial (*n* = 20) (Mann-Whitney test Z = 2.82, *asymp. p* = .005), and were slightly but not significantly more accurate in the Summation Identification trial (Z = 1.87, *asymp. p* = .061) (though note that few children (20%) responded incorrectly in the Summation Test trial, so these comparisons may be underpowered even with Experiments 1 and 2 combined).

In the Unknown Addend block, children’s Test success was related to their post-test accuracy: children who responded correctly in the Unknown Addend Test trial (*n* = 56) were more accurate than children who responded incorrectly in the Unknown Addend Test trial (*n* = 41) in both the Unknown Addend Comparison trial (Z = 2.55, *asymp. p* = .011) and Unknown Addend Identification trial (Z = 2.45, *asymp. p* = .014), Figure 4.

Across both blocks, there was no significant difference in age between children who responded correctly at Test compared to children who responded incorrectly (Summation: *t*(97) = 1.54, *p* = .127, Cohen’s *d* = .31; Unknown-addend: *t*(97) = 1.95, *p* = .054, Cohen’s *d* = .40).

### Discussion

In Experiment 2, we replicated the Summation condition from Experiment 1. Children were able to reliably solve for two sums in non-symbolic summation problems with small quantities, and they were able to use the solutions to those summation problems as inputs into a new, balancing operation. Children overall were not successful at solving for unknown addends when we controlled for lower-level strategies (i.e., by equating the final quantities between the two problems so that children could not use the relative size of the final quantities to support their responses on the Test trial). However, when children *did* successfully solve for the unknown addends, as evidenced by their responses in the post-test trials, they were able to use those solutions as inputs into a balancing operation. We discuss the implications for our understanding of the computational capacity of the parallel individuation system in the General Discussion.

## GENERAL DISCUSSION

In two experiments, we aimed to examine the computational capacity of the parallel individuation system by examining the kinds of operations children can do over individual object representations and whether children can compute with the outputs of such operations. We asked 4–6-year-old children to perform two types of non-symbolic exact arithmetic operations, summation operations in one block of trials, and unknown addend operations in another block of trials. After each block, we presented children with a Test trial in which they were shown two sets of objects with different quantities and asked children to balance the unequal sets by choosing which solution (generated from the summation or unknown addend problems) should be added to the smaller set to make the two sets the same. In Post-test trials following each Test trial, we directly probed children’s representations of the solution quantities. Here, we discuss three main takeaways from the results of these experiments.

First, when children are computing over small quantities of objects, computing sums is easier or more accessible than computing unknown addends (similar to previous results comparing these operations over AMS representations; Cheng & Kibbe, 2023a). There could be several reasons for this. One possibility is that computing subsequent additions of objects may just be more straightforward computationally than back-solving from a final quantity to determine what quantity must have been added to a set. Inferring the quantity of an unknown addend requires children to hold the initial set in working memory while observing the cup’s action, and then to perform a computation over the set held in working memory and the final set to derive their difference. There are several potential candidates for what this computation looks like. The computation could be something like a subtraction operation (which is supported by the parallel individuation system; Wynn, 1992), which children have to deploy over representations held in working memory (e.g., [observed final set] − [remembered set] = [addend]). The subtraction computation itself may be more challenging than the addition operation for children (Campbell, 2008; LeFevre et al., 2006), and the working memory demands of holding the first set in mind while performing the computation could also drive the differences in performance between summation and unknown addend formats. Or, the unknown addend computation could be carried out via a more complex backward inference process; children may reason counterfactually about *what must have been added* given the starting and final state of the set. Under this possibility, children may perform a sort of simulated incrementing operation (e.g., starting with a representation of one object and mentally incrementing the set until they get to the observed final quantity of three), which could impose higher cognitive demands and introduce more error compared to the forward incrementation that is possible over summation problems. Either potential computation would make unknown addend operations more challenging than summation operations, and further work is needed to better understand the exact computations supporting these two operations over individual object representations.

Another (non-mutually exclusive) reason that children may perform better at summation problems is that, while children of this age are still learning formal symbolic counting, ordinality, and arithmetic, they could be attempting to use verbal strategies to help support their problem solving in our task. For example, children might deploy a verbal “counting up” strategy in the summation problems, incrementing a verbal count list (albeit silently) as the objects are occluded. Such a strategy would be easier to deploy as children become more proficient with the count list, but more difficult to deploy over unknown addends, which would require children to directly compute the difference between counted sets. Perhaps as children acquire more experience with giving precise linguistic labels to their non-symbolic representations of small sets, they may be better able to represent and compare quantities and use those representations in a more general way, i.e., in other computations that are independent of the context in which the original result arose. However, we did not observe consistent age-related increases in children’s ability to solve for summation or unknown addend problems across Experiments 1 and 2 (as evidenced by their Test and Post-test responses). Further, previous work has shown that children in our age range consistently perform better on non-symbolically presented problems compared to similar verbally-presented problems (e.g., Levine et al., 1992), suggesting verbal strategies may be less reliable. We speculate that, while some children may have deployed verbally-mediated strategies, this is unlikely to be the only explanation for the differences in children’s performance across the summation and unknown addend problem contexts. Further work is needed to better pinpoint the sources that underly the difference in the computability and the role of verbal strategies for these problems for young children.

A second takeaway from Experiments 1 and 2 is that, while computing over unknown addends may be more challenging than computing sums, we observed that children who correctly identified the quantity of the unknown addend in the post-test trial were more successful at using the solved addends as inputs into a balancing operation, as evidenced by their Test trial performance. This suggests that some 4–6-year-old children can solve for unknown addends, in contrast to the conclusions of Kibbe and Feigenson (2017). Kibbe and Feigenson (2017) had a single dependent measure of children’s performance (their measure was similar to our Identification post-test trial), so they were not able to determine whether children’s chance-like performance was indeed due to chance or whether some children who chose correctly did so because they had successfully solved for the unknown addend. By including trials that asked children to compute with the solutions to the problems, we found that children of this age may show some success with this kind of computation, suggesting that the parallel individuation system may not be as computationally limited as previously thought. Further work is needed to better understand sources of individual differences in children’s ability to solve for unknown addends in small-quantity non-symbolic problems.

A third takeaway is, when children do solve for sums or unknowns in small-quantity non-symbolic arithmetic problems, they are able to use those solutions as inputs into a completely novel balancing operation. This contrasts with the results of Cheng and Kibbe (2023b) who found that while children were fairly precise in their representations of large unknown addends (suggesting that they solved for unknowns in large-quantity non-symbolic addend-unknown problems), they were not able to use those solutions as inputs into a balancing operation. Our results suggest that there may be more flexibility in the kinds of computations children can do over representations of smaller sets. We found that children are able to use small-quantity representations that did not arise from visual inputs—i.e., representations that resulted from an active operation over small-quantity representations—as an input into a new operation.

The contrast with Cheng and Kibbe’s (2023b) results also underscores the likelihood that children were using representations of small quantities, and not AMS representations, in our experiments. Cheng and Kibbe’s (2023b) study was set up similarly to our unknown addend conditions, except that they used larger sets of objects representable by the AMS. They found that 4–7-year-olds could solve for unknown addends quite successfully and with fairly high fidelity (see also Cheng & Kibbe, 2023a), but consistently failed to use those solutions as inputs into a new balancing operation. The results we obtained here suggest a qualitatively different pattern. Although the ratios between the initial and final quantities in the unknown addend problems we used in Experiments 1 and 2 were within the discriminability range for the AMS (and similar to the ratios used in Cheng & Kibbe, 2023b), children in our experiments overall struggled to solve for the unknowns, as evidenced by their Post-test trial performance. However, children in our experiments who showed positive evidence of solving for the unknown addends also showed evidence of being able to use those solutions as inputs into a new balancing operation, unlike the children in the large-set experiments of Cheng and Kibbe (2023b). While further experimental work is needed to more directly compare children’s performance across the small-large quantity divide, we think that the differences in patterns observed across these two papers is further suggestive of a computational difference between individual object representations and AMS representational formats.

We speculate that non-symbolic arithmetic over representations of small sets of objects may have a more “function-like” structure than non-symbolic arithmetic over representations of large sets (i.e., AMS representations). Symbolic arithmetic is defined by its function structure: any integer that is the result of an arithmetic computation is independent of the problem context in which it arose, and can therefore be used as input into any other problem (Dedekind & Beman, 1901; for further discussion, see Cheng & Kibbe, 2023b; Kibbe, 2023). Previous research by Cheng and Kibbe (2023b) suggested that non-symbolic arithmetic over AMS representations lacks this function-like structure. However, the results of the present research suggest that not all non-symbolic arithmetic operations are similarly computationally limited. We speculate that the representational format of small sets of objects may be more flexible in ways that would lend these representations to more function-like computation. Individual object representations are deployed separately from each other, are represented independently from each other, and are compared via one-to-one comparison of the individual representations. The independence of these representations that is inherent in their format may allow these representations to operate more flexibly across contexts. This format may also make individual object representations more readily transposed into other, more general, representational formats (like verbal number words) which could make them easier to compute with in a variety of contexts. However, further within-participants work is needed to better understand the contexts that give rise to different representational formats, the range of these different formats, the ways in which these formats and computations interface with symbolic number learning, and how differences in format may drive differential support for different computations across development, particularly as children become more adept at using symbolic counting, ordinality, and arithmetic strategies.

## ACKNOWLEDGMENTS

The authors would like to thank all children and their families who participated in our study, and Jiaqi Zhao, Shiba Esfand, Rakiya Washington, Vivien Jiang, and Emilia Boak for their assistance in data collection.

## FUNDING INFORMATION

This research was supported by a National Science Foundation (BCS 1844155) grant awarded to M. M. K.

## AUTHOR CONTRIBUTIONS

C.C.: Conceptualization: Equal; Formal analysis: Equal; Investigation: Lead; Methodology: Equal; Writing – original draft: Lead; Writing – review & editing: Equal. M.M.K.: Conceptualization: Equal; Formal analysis: Equal; Funding acquisition: Lead; Investigation: Supporting; Methodology: Equal; Writing – original draft: Supporting; Writing – review & editing: Equal.

## DATA AVAILABILITY STATEMENT

Stimuli, data, and an example video demonstration of study procedures are available in the Open Science Framework at https://osf.io/jkc5f/.
